# A high-dimensional immune monitoring model of HIV-1-specific CD8 T cell responses accurately identifies subjects achieving spontaneous viral control

**DOI:** 10.1186/1742-4690-9-S2-P280

**Published:** 2012-09-13

**Authors:** ZM Ndhlovu, L Chibnik, J Proudfoot, S Vine, A McMullen, K Cesa, F Porichis, D Alvino, A Piechocka-Trocha, P De Jager, BD Walker, D Kaufmann

**Affiliations:** 1 Ragon Institute of MGH, MIT and Harvard, Charlestown, MA, USA; 2 Brigham and Women's Hospital & Harvard Medical School, Boston, MA, USA

## Background

The development of immune monitoring models to determine HIV-1 vaccine efficacy is a major challenge. HIV-1-specific CD8 T cells likely play a critical role in individuals achieving spontaneous viral control (HIV-1 controllers) and will be important in immune interventions. However, no single CD8 T cell function is uniquely associated with controller status.

## Methods

Here we describe the building of immune monitoring models based on inter- and intra-donor analysis of HIV-1-specific CD8 T cell proliferation and cytokine secretion assessed at different time points after antigen stimulation. The discovery data set used to build a palette of immune monitoring models of HIV-1-specific CD8 T cell functions was generated on 26 controllers, 15 progressors and 23 ART-treated subjects. An independent cohort of 10 controllers and 10 progressors was investigated to validate our results. We used Area Under the Receiving Operating Characteristic curves (AUC) to assess the ability of individual variables to differentiate between controllers and non-controllers.

## Results

Our analyses identified links between HIV-1-specific CD8 T functions, HLA-I alleles, and disease stage. The best accuracy (AUC) values were observed for proliferation. Early (6h) IL-2 secretion and slopes of TNF-α, IL-2 and IFN-γ production also contributed to the models whereas gender and age had no discriminatory value. A model incorporating five HIV-1-specific CD8 T cell functions achieved 90% accuracy in the discovery cohort on which it was trained, and was able to accurately discriminate controllers from non-controllers in the validation cohort.

## Conclusion

Our multidimensional modeling approach shows that integration of different dimensions of data leverages independent associations and discriminates much better than any one measure. This modeling approach is amenable to incremental incorporation of new knowledge to build evolving flexible tools that are usable in translational clinical research and thus, has important applications in predictive model development and immune monitoring of HIV-1 vaccine trials.

